# Fatty Acid Transport Protein 1 (FATP1) Localizes in Mitochondria in Mouse Skeletal Muscle and Regulates Lipid and Ketone Body Disposal

**DOI:** 10.1371/journal.pone.0098109

**Published:** 2014-05-23

**Authors:** Maria Guitart, Óscar Osorio-Conles, Thais Pentinat, Judith Cebrià, Judit García-Villoria, David Sala, David Sebastián, Antonio Zorzano, Antonia Ribes, Josep C. Jiménez-Chillarón, Celia García-Martínez, Anna M. Gómez-Foix

**Affiliations:** 1 Departament de Bioquímica i Biologia Molecular, Facultat de Biologia, Universitat de Barcelona (UB), Institut de Biomedicina de la UB, Barcelona, Spain; 2 Hospital Sant Joan de Déu, Endocrinology, Esplugues, Barcelona, Spain; 3 Sección de Errores Congénitos del Metabolismo (IBC), Servicio de Bioquímica y Genética Molecular, Hospital Clínico, Institut d'Investigacions Biomèdiques August Pi i Sunyer, Barcelona, Spain; 4 CIBER de Enfermedades Raras (CIBERER), Instituto de Salud Carlos III, Spain; 5 Institute for Research in Biomedicine, Barcelona, Spain; 6 CIBER de Diabetes y Enfermedades Metabólicas Asociadas (CIBERDEM), Instituto de Salud Carlos III, Spain; 7 Departament de Patologia i Terapèutica Experimental, UB, Hospitalet de Llobregat, Barcelona, Spain; INSERM/UMR 1048, France

## Abstract

FATP1 mediates skeletal muscle cell fatty acid import, yet its intracellular localization and metabolic control role are not completely defined. Here, we examine FATP1 localization and metabolic effects of its overexpression in mouse skeletal muscle. The FATP1 protein was detected in mitochondrial and plasma membrane fractions, obtained by differential centrifugation, of mouse gastrocnemius muscle. FATP1 was most abundant in purified mitochondria, and in the outer membrane and soluble intermembrane, but not in the inner membrane plus matrix, enriched subfractions of purified mitochondria. Immunogold electron microscopy localized FATP1-GFP in mitochondria of transfected C2C12 myotubes. FATP1 was overexpressed in gastrocnemius mouse muscle, by adenovirus-mediated delivery of the gene into hindlimb muscles of newborn mice, fed after weaning a chow or high-fat diet. Compared to GFP delivery, FATP1 did not alter body weight, serum fed glucose, insulin and triglyceride levels, and whole-body glucose tolerance, in either diet. However, fatty acid levels were lower and β-hydroxybutyrate levels were higher in FATP1- than GFP-mice, irrespective of diet. Moreover, intramuscular triglyceride content was lower in FATP1- versus GFP-mice regardless of diet, and β-hydroxybutyrate content was unchanged in high-fat-fed mice. Electroporation-mediated FATP1 overexpression enhanced palmitate oxidation to CO2, but not to acid-soluble intermediate metabolites, while CO2 production from β-hydroxybutyrate was inhibited and that from glucose unchanged, in isolated mouse gastrocnemius strips. In summary, FATP1 was localized in mitochondria, in the outer membrane and intermembrane parts, of mouse skeletal muscle, what may be crucial for its metabolic effects. Overexpressed FATP1 enhanced disposal of both systemic fatty acids and intramuscular triglycerides. Consistently, it did not contribute to the high-fat diet-induced metabolic dysregulation. However, FATP1 lead to hyperketonemia, likely secondary to the sparing of ketone body oxidation by the enhanced oxidation of fatty acids.

## Introduction

The cellular uptake of long-chain fatty acids is known to be largely protein mediated and several protein families have been involved in this process. One of these families is the fatty acid transport protein (FATP), currently with six members identified in mammalian genomes (FATP1–6) [1], [2]. One of these family members is the FATP1 gene, which is expressed at high levels in skeletal muscle (skm), heart and adipose tissue, and at low levels in brain, kidney, lung and liver in mice [3].

FATP1 is an integral membrane protein with one transmembrane domain in the amino terminus region of the protein [4] and displays intrinsic acyl-CoA synthetase activity, which is nevertheless lower relative to other fatty acid CoA ligases, such as FATP4 and acyl-CoA synthetase long-chain family member 1 (ACSL1) [5]. FATP1 has been localized in the plasma membrane of differentiated 3T3-L1 adipocytes [3], [6], [7], or insulin-stimulated 3T3-L1 adipocytes [6], [7], and 293 cells [8]; however, FATP1 has consistently been found in intracellular compartments of adipocytes and muscle cells in culture. In 3T3-L1 adipocytes, it was found in a perinuclear compartment overlapping with a Golgi marker in non-stimulated cells [7], and in mitochondria [9]; in another study, FATP1 was localized in the endoplasmic reticulum but not in mitochondria [10]. In cultured human myotubes, we showed that FATP1 is not present in the plasma membrane, but intracellularly, in a reticular and perinuclear pattern, partially overlapping with a Golgi marker [11]. Furthermore, we localized FATP1 in the mitochondria-enriched fractions of cultured human and C2C12 muscle cells [12]; and co-localized a tagged FATP1-GFP fusion protein with mitochondrial markers in both C2C12 [12] and L6E9 [13] muscle cells. Sarcolemmal staining and pronounced intracellular FATP1 localization in an undefined vesicle population has been observed in isolated mouse soleus muscle [14], as well as the presence of FATP1 in the t-tubule and sarcolemma fractions of lower hindlimb rat muscles [15]. However, no evidence was obtained for the localization of transfected FATP1 on mitochondrial membranes in mature rat skm [16].

FATP1 is able to enhance fatty acid uptake in cultured skm cells [11] and in rodent muscle tissue [16]. However, on the basis of its subcellular localization, it is argued whether FATP1-mediated cell fatty acid import is due to transbilayer movement of fatty acids in the plasma membrane or to a driving force associated with its intrinsic acyl-CoA synthetase, which might trap the entering fatty acids as acyl-CoAs [10], [17] and direct its metabolism. In fact, our studies in cultured skm cells showed that FATP1 targets fatty acids towards triacylglycerol synthesis [11], [13], whereas fatty acid oxidation is either moderately stimulated [13] or slightly reduced [11]. In contrast, a previous study [16] addressing the role of FATP1 in rodent muscle metabolic control, by means of its overexpression, showed different fatty acid targeting, i.e. electrotransfection of FATP1 into skm of rats increases fatty acid oxidation but not triacylglycerol synthesis in isolated muscle preparations; while muscle-specific overexpression of FATP1 in transgenic mice elevates the rate of *in vivo* fatty acid uptake in skm of mice on a chow diet, but not of high-fat diet-induced intramuscular triglyceride accumulation. It was concluded that FATP1 does not promote mouse intramuscular triglyceride accumulation and whole body insulin resistance [16]. In addition to fatty acid metabolism, FATP1 is also able to enhance glucose metabolism in cultured skm cells, in which it exerts a powerful stimulation of glucose oxidation and activates the pyruvate dehydrogenase (PDH) complex [12].

Genetic ablation of FATP1 in mice does not change basal fatty acid uptake, but reduces its stimulation by insulin, in isolated muscle strips and delays the insulin-induced clearance of serum fatty acids [14]. Moreover, abrogation of FATP1 gene protects mice from the fat-induced accumulation of fatty acyl-CoA and fat-induced insulin resistance in skm [18]. In fact, FATP1 gene knockout in mice protects against high-fat diet-induced reduction of insulin-stimulated whole-body glucose turnover [18]; and impairment of insulin tolerance and signs of metabolic syndrome [14]. In this regard, FATP1 inhibition is a therapeutic target for insulin resistance [19].

The aim of this work was to gain new insight into the subcellular localization and role of FATP1 in metabolic control in skm tissue. Thus, we examined the possible mitochondrial localization of FATP1 in rodent skm and conducted new investigations on the localization of FATP1 in the mitochondria of C2C12 muscle cells. Moreover, we overexpressed FATP1 in gastrocnemius mouse muscle, by means of adenovirus or electroporation, to test its effects on skm lipid metabolism and the expression of key control genes in ketone body metabolism and glucose versus fatty acid disposal, on a normal chow diet and following fat-induced metabolic dysregulation.

## Materials and Methods

### Ethics Statement

The animal protocols were approved by the Universitat de Barcelona Animal Care and Use Committee (Permit number: DMAH-5444). Animal sacrifice was performed under CO_2_ anesthesia.

### Gene transfer methods, cell culture and animals

Plasmids pGFP (pEGFP-N1) and p-FATP1-GFP [11] were used. The C2C12 cell line was grown in DMEM medium with 10% FBS and was induced to differentiate in DMEM with 10% horse serum for 4 days. The C2C12 cell line [20] was transfected with plasmids pGFP and/or p-FATP1-GFP by means of GeneJuice (Merck Millipore, Darmstadt, Germany).

Recombinant adenoviruses expressing the cDNA of mouse FATP1 (Ad-FATP1) or GFP (Ad-GFP) have been described previously [11]. For delivery to mice, adenoviruses produced in 293 cells were purified using the ViraBind purification kit (Cell Biolabs, San Diego, USA) and purified virus preparations were desalted using Microspin G-25 columns. Two separate batches of littermates, 11 males and 30 females, were used. Newborn (5 days old) C57BL6 male and female mice were injected in the gastrocnemius muscle in both legs with approximately 2×10^8^ plaque forming units (pfu) (injected volume was less than 10 µl) of Ad-GFP (5 males and 14 females) or Ad-FATP1 (6 males and 16 females), as described in [21]. Mice were weaned at 21 days. Thereafter, mice were randomly allocated to two dietary treatments: a standard laboratory chow diet (2014 Tekland Global, Harlan Iberica, Barcelona, Spain) (11 males and 10 females) or a high-fat diet (DIO rodent purified diet with 45% energy from fat, TestDiet, Richmond, USA) (20 females); and water *ad libitum*. When stated, animals were fasted by 15 h-deprivation of food. Animals were sacrificed at 15- to 16-weeks of age in the fed state. Blood was collected and serum was stored at −20°C. Tissues were excised and immediately frozen in liquid N_2_ and stored at −80°C. When stated tissue samples were powdered in a mortar under liquid N_2_ and thereafter homogenized.

Plasmids pGFP (pACCMV-GFP) or p-FATP1 (pACCMV-FATP1) [11] were used for electrotransfer studies. Plasmids were purified and resuspended in Endofree water (Qiagen, Crawley, UK) and dissolved in 0.9% NaCl. Three month-old mice (C57BL6/J) were anesthetized with ketamine/xylazine and 1 h before electrotransfer muscles were pretreated with hyaluronidase (10 U/muscle). Afterwards, 60 µg of pGFP or pFATP1 were injected into the gastrocnemius muscle of both hindlimbs of 3 mice. Ten pulses of 20 ms each were applied to each hindlimb at 175 V/cm and 1 Hz using an electroporator (ECM 830; BTX, Holliston, USA). Mice were provided with standard laboratory chow diet. Animals were killed 10 days after the electrotransfer and gastrocnemius muscles were excised and immediately submerged in DMEM without glucose plus 0.1 mM free fatty acid BSA and 15 mM Hepes. Gastrocnemius strips were obtained by dissection with the aid of scissors and forceps with a modification of the protocol in [22]. Fat and connective tissue were removed under the scope. Strips of 20 to 25 mg wet weight were prepared (about 10 to 12 strips per muscle) and were then randomly mixed to account for regional differences in fiber type distribution [23]. Two strips were immediately frozen to determine FATP1 protein content and the rest of the strips were incubated for metabolic assays.

### RNA extraction, reverse transcription and real-time PCR

Total RNA was extracted from tissue samples using the RNeasy minikit (Qiagen, Valencia, CA, USA) and homogenized using a Polytron (Kynematica Polytron, Westbury, USA). Total RNA (0.5 µg) was retro-transcribed (RT) using TaqMan reverse transcription reagents from Applied Biosystems (Branchburg, USA) plus random hexamers. Real-time PCR was performed using the ROCHE sequence detection system with the TaqMan universal PCR master mix and probes (Applied Biosystems) for the mouse Slc27a1/FATP1, Oxct1 and Pdk4 genes, using *Rn18s* as the endogenous control to normalize the crossing point (CP). For the Hmgcs2 gene real-time PCR was performed using the ABI PRISM 7500 sequence detection system (Applied Biosystems) with the GoTaq qPCR master mix (Promega) and SyberGreen labelling and using the Actb gene as the endogenous control to normalize the threshold cycle (CT).

### Preparation of muscle extracts, isolation of pure mitochondria by Percoll gradient and submitochondrial fractionation

To prepare extracts from skm tissue, 25 mg of frozen gastrocnemius muscle was crushed into small pieces and homogenized in 0.5 ml of buffer (0.25 M sucrose, 1 mM EDTA, 10 mM HEPES (pH 7.4), 0.5 mM PMSF and 1 U/ml aprotinin) using a glass homogenizer (20 to 25 strokes). Extracts were homogenized with the aid of a 22 G syringe. An aliquot of extracts was centrifuged at 500 *g* for 5 min at 4°C and supernatants kept for further analysis (total extracts). When stated, the homogenate was centrifuged at 1500 *g* for 10 min at 4°C to obtain a pellet, highly enriched in large mitochondria that also contained nuclei [24], which was re-suspended in 60 µl of homogenization buffer. The supernatant was further centrifuged at 10000 *g* for 10 min at 4°C, to obtain another pellet enriched in mitochondria and other organelles; the supernatant was kept and the pellet was re-suspended in 60 µl of homogenization buffer.

Isolation of pure mitochondria from four gastrocnemius from two mice was performed as described in Wieckowski et al. [25] with some modifications. Muscles were washed in ice-cold IB-1 buffer (225 mM mannitol, 75 mM sucrose, 0.5% BSA, 0.5 mM EGTA and 30 mM Tris-HCl (pH 7.4)), cut into small pieces using scissors and homogenized using first a polytron and then a glass homogenizer in a ratio of 4 ml IB-1 buffer per gram of muscle. The homogenate was centrifuged at 740 *g* for 5 min at 4°C and the supernatant was centrifuged again in the same conditions. The resulting supernatant was then centrifuged at 9000 *g* for 10 min at 4°C. The obtained mitochondria enriched pellet was resuspended in 2 ml of ice-cold IB-2 buffer (225 mM mannitol, 75 mM sucrose, 0.5% BSA and 30 mM Tris-HCl (pH 7.4)) and the supernatant was kept. An aliquot of this supernatant was ultracentrifuged at 100000 *g* for 60 min at 4°C. The resulting pellet designated “membrane fraction” (containing plasma membrane, lysosomes, microsomal fraction and large polyribosomes) was resuspended in 20 µl of buffer (0.25 M sucrose, 1 mM EDTA, 10 mM HEPES (pH 7.4), 0.5 mM PMSF and 1 U/ml aprotinin). The resuspended mitochondria enriched pellet was centrifuged at 10000 *g* for 10 min at 4°C, the obtained pellet was resuspended in 2 ml of ice-cold IB-3 buffer (225 mM mannitol, 75 mM sucrose and 30 mM Tris-HCl (pH 7.4)) and centrifuged at 10000 *g* for 10 min at 4°C. The final crude mitochondrial pellet was resuspended in 2 ml of MRB buffer (250 mM mannitol, 5 mM HEPES (pH 7.4) and 0.5 mM EGTA) and was then layered on top of Percoll medium (8 ml Percoll medium, 2 ml of crude mitochondria and 3.5 ml of MRB buffer) and centrifuged at 95000 *g* for 30 min at 4°C. After centrifugation, purified mitochondria appeared as a brownish band at the bottom of the tube. Pure mitochondria was collected by using a Pasteur pipette, diluted 10 times in MRB buffer and centrifuged at 6300 *g* for 10 min at 4°C. Mitochondrial pellet was resuspended in 2 ml of MRB buffer and centrifuged again at 6300 *g* for 10 min at 4°C. The pellet, containing purified mitochondria, was resuspended in 200 µl of MRB buffer and stored at −20°C.

Part of the purified mitochondria obtained as indicated above were used to separate three fractions: inner membrane plus matrix, outer membrane and a soluble fraction (containing proteins localized between the membranes plus some solubilized outer membrane), as described in [26], [27] with some modifications. One hundred µg of purified mitochondria were treated with digitonin (2% solution in 250 mM sucrose) at a ratio of 1.2 mg of digitonin per 10 mg of mitochondrial protein. The resulting suspension was gently stirred for 15 min and then diluted with 3 volumes of a mannitol solution (210 mM mannitol plus 70 mM sucrose and 0.1 mM EDTA). The diluted suspension was centrifuged at 12000 *g* for 12 min at 4°C. The supernatant was carefully drawn off, and the pellet was gently resuspended in the same volume of the mannitol solution. This suspension was centrifuged again at the same speed for 12 min. The pellet from the second centrifugation was designated as the “inner membrane plus matrix fraction.” The supernatants from the first and second centrifugation were pooled and further centrifuged at 105000 *g* for 90 min at 4°C. The pellet from this centrifugation was designated as the “outer membrane fraction,” and the supernatant solution as the “soluble fraction.”

### Western blotting

Protein was resolved in 10% SDS-PAGE and immunoblotting was performed with antibodies against FATP1, SCOT/OXCT1, HMGCS2 and oxidoreductase-protein disulfide isomerase (PDI) (Santa Cruz Biotechnology, Santa Cruz, USA); porin/voltage dependent anion channel (VDAC) and glyceraldehyde 3-phosphate dehydrogenase (GAPDH) (Cell Signalling Technology, Beverly, USA); α-actinin (Chemicon International, Temecula, USA), α-tubulin (Sigma-Aldrich, St. Louis, USA), glucose transporter GLUT1 (Abcam, Cambridge, UK) and oxphos complex IV subunit I (mitochondrially encoded cytochrome c oxidase I (MTCO1)) (Life Technologies, Carlsbad, USA); and phosphorylation sites 1 and 2 of pyruvate dehydrogenase-E1α (kindly provided by Dr. H. Pilegaard [28]). Horseradish peroxidase-conjugated secondary antibodies were used and membranes were developed with ECL-Plus (GE Healthcare, Buckinghamshire, UK). Protein bands were revealed and quantified using a LAS-3000 luminescent image analyzer (FujiFilm, Tokyo, Japan).

### Transmission electron microscopy

Cultured cells were chemically fixed at 4°C with a mixture of 2% paraformaldehyde and 0.1% glutaraldehyde in phosphate buffer (PB) (pH 7.2). After washing with PB containing 50 mM glycine, cells were embedded in 12% gelatin and infused in 2.3 M sucrose. Mounted gelatine blocks were frozen in liquid nitrogen. Sections were prepared using an ultracryomicrotome (Leica EM Ultracut UC6/FC6, Vienna, Austria). Ultrathin cryosections were collected with 2% methylcellulose in 2.3 M sucrose. Cryosections were successively incubated at 37°C on drops of 2% gelatin in PBS for 20 min, 50 mM glycine in PBS for 15 min, 10% FBS in PBS for 10 min, and 5% FBS in PBS for 5 min. Then, they were incubated with anti-GFP antibody (Life Technologies) in 5% FBS in PBS for 30 min. After three washes with drops of PBS for 10 min, sections were incubated for 20 min using protein A coupled to 10 nm diameter colloidal gold particles (Cell Microscopy Center, Department of Cell Biology, University Medical Center Utrecht, The Netherlands), using a 1∶60 dilution in 5% FBS/PBS. This was followed by three washes with drops of PBS for 10 min, and two washes with distilled water. As a control for non-specific binding of the colloidal gold-conjugated protein A, the primary polyclonal antibody was omitted. Observations were made in a Jeol J1010 Electron Microscope (Jeol, Tokyo, Japan) with a SIS Megaview III CCD camera.

### Metabolite and hormone assays

Insulin was measured in serum using mouse insulin ELISA (Merck Millipore). Serum triglycerides were measured using a colorimetric method (Biosystems, Barcelona, Spain). β-Hydroxybutyrate determination was performed in serum using the Cayman β-Hydroxybutyrate Assay kit (Cayman Chemical, Ann Arbor, USA). Serum free fatty acids were measured using a Quantification Kit (BioVision, Mountain View, USA). Blood glucose was determined using a Glucometer Elite (Menarini, Barcelona, Spain).

For triglyceride assays in tissue samples, 15 mg of frozen powdered tissue were homogenized in a buffer containing 50 mM Tris-HCl (pH 7.9), 100 mM KCl, 20 mM KF, 0.5 mM EDTA and 0.05% lubrol. The resulting extracts were sonicated (three times for 10 s at 15 s intervals), centrifuged at 11000 *g* for 15 min at 4°C, and the supernatants were used for the determinations. Triglycerides were measured using a colorimetric method (Biosystems). The protein content of individual samples was determined using the Pierce BCA protein assay kit (Thermo Scientific, Rockford, USA).

For β-hydroxybutyrate tissue determination, 30 mg of frozen powdered tissue were homogenized in 600 µl of distilled water using a Polytron, centrifuged at 800 *g* for 10 min and the supernatants collected. Organic acids from 500 µl of supernatants were analyzed by gas chromatography-mass spectrometry as TMS-derivates, as previously described [29], but using 7 µl of 5 mM undecanodioic acid as the internal standard and SIM mode monitoring ions 191 and 345 for the detection of β-hydroxybutyric and undecanodioic acids respectively.

### Glucose and insulin tolerance test

The glucose tolerance test was performed on 14-week-old conscious mice after an overnight fast. On the following day the mice were injected intraperitoneally with a glucose bolus of 2 g/kg body weight in a volume of no more than 200 µl. Glucose was monitored before and 15, 30, 60 and 120 min after glucose administration. The area under the curve of glucose concentration was calculated using the trapezoidal rule. Animals were fed after the test. The insulin tolerance test was performed on 15-week-old conscious mice after 4 h fasting. The animals were provided with water *ad libitum*. An insulin bolus (1 unit/Kg of body weight) was administered by intraperitoneal injection. Glucose level was monitored via the tail before and 15, 30 and 60 min after insulin administration. Animals were re-fed after the test.

### Pyruvate dehydrogenase assay

Active pyruvate dehydrogenase (PDH) activity was determined by measuring the ^14^CO_2_ production from [1-^14^C]-pyruvate according to the method described by Van Laack et al. [30], with modifications. Tissue extracts were prepared by manually homogenizing 15 mg of frozen and powdered skm into 300 µl of buffer, composed of 250 mM sucrose, 2 mM EDTA, 100 UI/ml heparin and 10 mM Tris (pH 7.4), using 20 to 25 strokes of a glass pestle. The homogenate was centrifuged for 10 min at 800 *g* and the supernatant was collected. Twenty-five µl of tissue extracts were preincubated at 37 °C with 25 µl of an assay mixture containing 400 mM Tris-HCl (pH 7.8), 2 mM EDTA, 8 mM MgCl_2_, 3 mM TPP, 2 mM NAD, 8 mM CoA, 4 mM L-carnitine, 80 µM cytochrome c, 20 mM β-mercaptoethanol, 4 mM Na_2_SO_3_ and 5 µl neonatal calf serum. After 10 min of preincubation, 25 µl of 2 mM [1-^14^C]-pyruvate (0.7 µCi/µmol) was added and the reaction continued at 37°C for 15 min in tubes placed in closed vials. The reaction was stopped by injecting 200 µl of 20% trichloroacetic acid into the tubes. The ^14^CO_2_ released was trapped in 1 cm^2^ filter papers, soaked with 0.5 M NaOH and stuck to the vial wall, for 30 min. The radioactivity in filter papers was counted, in a liquid scintillation counter, and values corrected for protein concentration, as measured with a Pierce BCA protein assay kit, to calculate PDH activity. To examine the levels of phosphorylated PDH-E1α subunit at sites 1 and 2, aliquots of tissue extracts (25 µg of protein) were analysed by western blotting.

### Assay of substrate oxidation in isolated gastrocnemius muscle strips

Two to 3 strips of mouse gastrocnemius muscle (total of 50 to 60 mg) were dispensed into plastic vials and incubated during 4 h (37°C, 5% CO_2_) in 2.5 ml of glucose-depleted DMEM medium containing either 10 mM glucose added with [U-^14^C]glucose (0.21 µCi/µmol) (Amersham Biosciences, Barcelona, Spain), 0.5 mM palmitate added with [1-^14^C]palmitate (2.8 µCi/umol) (Amersham Biosciences) or 2 mM β-hydroxybutyrate added with p-[3-^14^C]hydroxybutyrate (0.2 µCi/µmol) (NEN Research Products, DuPont, Boston, USA). An aliquot of 10 µl of the incubation media was taken before and at the end of muscle strips incubation, and was counted for radioactivity in scintillation cocktail (Ecoscint H, National Diagnostics, East Riding of Yorkshire, UK) to estimate substrate utilization rate during the incubation period. The incubation was terminated by addition of 750 µl of 3 M HClO_4_ per vial. Immediately, a Whatman #3 paper soaked with 125 µl β-phenylethylamine was placed hanging in a basket in each vial tightly sealed with a silicone cap. After 1 h of incubation the filter paper was harvested and the trapped ^14^CO_2_ counted in scintillation cocktail. In muscle strips incubated with [1-^14^C]palmitate, [^14^C]acid-soluble intermediate metabolites were determined by harvesting 200 µl of incubation media added with HClO_4_, centrifuging this at 13000 *g* for 5 min (to discard acid insoluble long-chain fatty acids), collecting 100 µl of the resulting supernatants (containing oxidized fatty acid intermediate metabolites) and measuring the radioactivity of these in scintillation cocktail.

### Statistical analysis

Data are presented as means ± SEM or SD. The significance of differences was analyzed by Student's t test. Differences were considered significant at p<0.05. Student's t test was applied to the subgroups defined by gene delivery, gender and diet; or was applied to the groups defined by one condition, such as gene delivery, when no significant differences were observed within the other conditions, such as gender and/or diet.

## Results

### Mitochondrial localization of FATP1 protein in mouse skm and cultured C2C12 muscle cells

**Figure 1:**
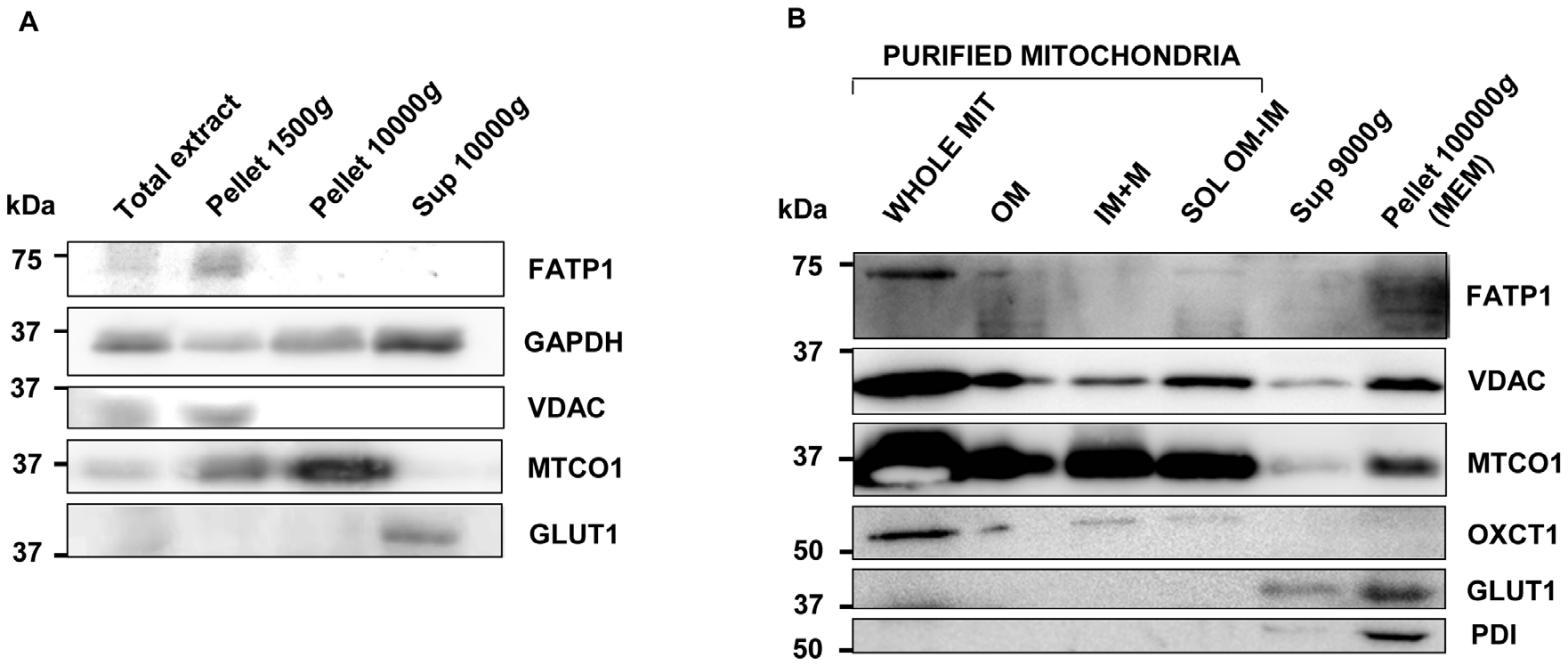
FATP1 protein is localized in mitochondria in gastrocnemius muscle. Gastrocnemius muscle samples of adult control mice were used and western blot analyses were performed on tissue extracts. (A) Thirty µg of protein of total extracts, pellets after centrifugation at 1500 *g* and 10000 *g* or the supernatant (sup) of the 10000 *g* pellet. (B) Extracts from purified mitochondria, namely whole mitochondria (WHOLE MIT) (20 µg of protein), subfractions of outer membrane (OM) (10 µg of protein), inner membrane plus matrix (IM+M) (10 µg of protein) and soluble intermembrane protein (SOL OM-IM) (3 µg of protein); the 9000 *g* supernatant of pelleted mitochondria (20 µg of protein) and the 100000 *g* pellet membrane (MEM) of the 9000 *g* supernatant (20 µg of protein). Membranes were hybridized with antibodies against FATP1, VDAC, MTCO1, GAPDH, OXCT1, GLUT1 or PDI as indicated. Representative images are shown.

We have previously reported that endogenously expressed FATP1 in cultured human and C2C12 muscle cells is present in mitochondria-enriched fractions and not in the cytosolic fraction [12]. Here, we examined by immunoblotting the distribution of endogenous FATP1 protein in subcellular fractions of skm from adult mice fed a normal diet obtained by sequential centrifugation (Figure 1A). VDAC and MTCO1 were used as mitochondrial markers, GAPDH as a cytoplasmic predominant protein and GLUT1 as a plasma membrane predominant protein. As shown in Figure 1A, FATP1 (about 71 kDa) was detected in the 1500 *g* pellets, which are enriched in large mitochondria [24], and not in the 10000 *g* pellets, which are enriched in mitochondria and other organelles, or in the 10000 *g* supernantant fraction. The FATP1 distribution was equivalent to that of the mitochondrial marker VDAC [31]. It was however different from that of MTCO1, a component of the respiratory electron transport chain of mitochondria, which was present (observed size 37 kDa) in both 1500 *g* and 10000 *g* pellets, but predominant in the 10000 *g* pellet. FATP1 distribution differed from that of GAPDH, which is known to be localized mainly in cytoplasm, although it can also be found in particulate fractions, such as the nucleus, the mitochondria, and the small vesicular fractions [32]. FATP1 distribution differed from that of GLUT1, which was present in the 10000 *g* supernatant fraction. Therefore, these data showed the preferential distribution in the mouse skm mitochondrial fractions of the endogenous FATP1 protein.

Then, we applied a density gradient centrifugation technique to purify mitochondria from the mitochondrial pellet. The analysis of FATP1 protein presence in purified mitochondria is shown in Figure 1B. FATP1 was strongly detected in purified mitochondria and hardly detected in the mitochondrial pellet supernatant. The purity of mitochondria was highlighted by the lack of staining with the endoplasmic reticulum resident oxidoreductase chaperone PDI and plasma membrane marker GLUT1, in opposition to the marked presence of mitochondrial proteins such as VDAC, MTCO1 and OXCT1. PDI and GLUT1 were shown in the supernatant of the mitochondrial pellet, even though mitochondrial proteins VDAC and MTCO1 were also detected in this fraction.

Purified mitochondria were then treated with digitonin, to remove outer membranes, followed by differential centrifugation to separate three fractions enriched in: the outer membrane, the soluble proteins (including proteins localized between the membranes plus some solubilized outer membrane) or the inner membrane plus matrix [26]. FATP1 appeared in the outer membrane and soluble fraction (despite the latter contained about 3-fold less protein than the other two submitochondrial fractions), but not in the inner membrane plus matrix fraction (Figure 1B). The distribution pattern of VDAC, a protein known to be localized on the outer mitochondrial membrane, was similar with predominance in the outer membrane and soluble fractions; although a signal was present in the inner membrane plus matrix fraction. In contrast, the mitochondrial matrix enzyme OXCT1 and the mitochondrial-encoded MTCO1, which is localized within the mitochondrial inner membrane, were most abundant in the inner membrane plus matrix enriched fraction; although they were also detected in the other two fractions, mainly the soluble one.

We then examined FATP1 presence in a membrane fraction (plasma membrane and microsomes) obtained by ultracentrifugation of the mitochondrial pellet supernatant. As shown in Figure 1B, FATP1 was detected in this part. The endoplasmic reticulum maker PDI and the plasma membrane protein GLUT1 were most abundant in this fraction as expected. VDAC, which has been demonstrated to have also a plasma membrane localization [33], was shown at a considerable level. A very low proportion of MTCO1 and no signal for the mitochondrial matrix protein OXCT1 was observed.

**Figure 2:**
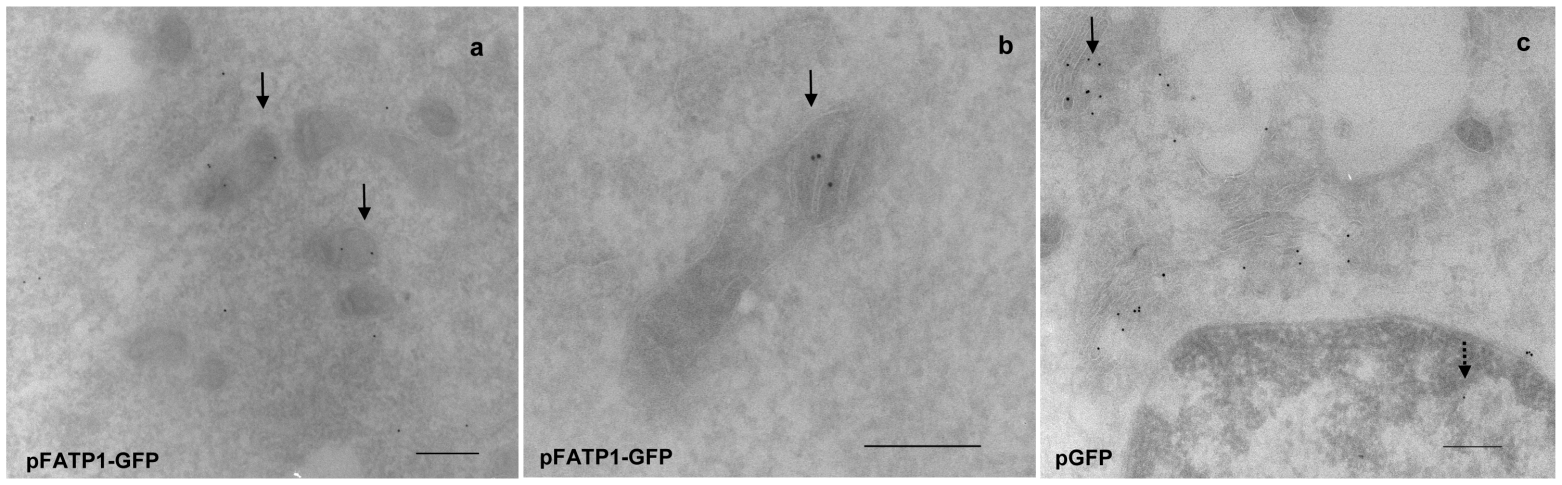
Electron microscopic localization of FATP1-GFP in C2C12 myotubes. C2C12 myoblasts were transfected with (a,b) pFATP1-GFP or (c) pGFP (control), and induced to differentiate into myotubes. Four days post-transfection, myotubes were fixed, gelatin blocks mounted and sections were prepared in an ultracryomicrotome, incubated with anti-GFP antibody and analyzed by electronic microscopy. (a,b) Image of FATP1-GFP localized inside the mitochondria (see arrows); (c) image of GFP localized in the Golgi complex (see continuous arrows) and nuclei (dotted arrow). The observations were made in an electron microscope with a CCD camera and an electron accelerating voltage of 80 Kv was employed for the measurements. Bar represents 200 nm.

Previously, applying fluorescence microscopy we showed that transfected FATP1-GFP colocalized with mitochondrial markers in C2C12 [12] and L6E9 [13] cells. Here, to achieve higher resolution in the study of the subcellular localization of FATP1 in muscle cells, we performed an immunocytochemical analysis of the transfected FATP1-GFP or GFP encoded proteins in C2C12 muscle cells with an anti-GFP antibody by electron microscopy (Figure 2). This showed that the FATP1-GFP protein was localized in the mitochondria in C2C12 myotube sections (Figure 2a,b), in addition to other cytoplasmic locations. In contrast, the GFP protein (Figure 2c) was mainly localized in the Golgi complex and it was present inside the nuclei, but not found within mitochondria.

### Adenovirus-mediated FATP1 overexpression in mouse gastrocnemius muscle

**Figure 3:**
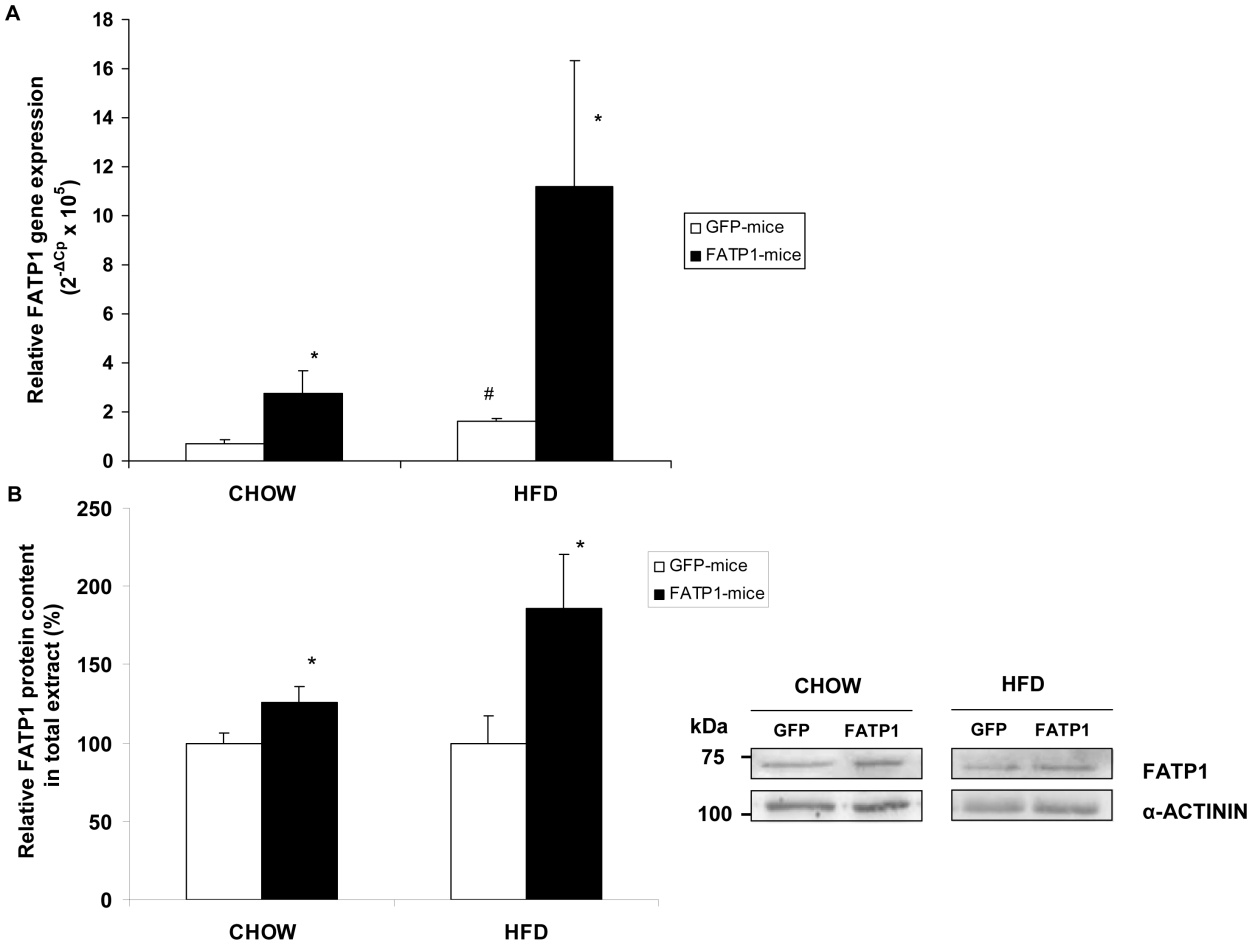
Overexpression of the FATP1 gene in mouse gastrocnemius muscle. (A) The FATP1 mRNA levels relative to *Rn18s*, and (B) the FATP1 protein levels relative to α-actinin, were analyzed in the gastrocnemius muscles (right and left) of GFP- and FATP1-mice fed a chow diet (males and females) or a high-fat diet (HFD) (females). (A) Data are mean values of 2^−ΔCp^×10^5^ ± SEM from nine to sixteen samples performed in duplicate. (B) A representative image is shown and bands were quantified. Data is the ratio of intensities of bands expressed as a percentage of control and is the mean ± SEM of five samples performed in duplicate. The significance of the Student's t test is: ^#^p<0.01 GFP-females fed a HFD versus GFP-mice (male and female) fed a chow diet; and *p<0.05 FATP1- versus GFP-mice within the same diet condition.

To bring new insight into the impact of skm FATP1 on metabolic control, the FATP1 or the GFP coding sequences were delivered into newborn mice by intramuscular injection of adenovirus Ad-FATP1 (FATP1-mice), or Ad-GFP (GFP-mice), into both hind legs (to maximize the whole body metabolic impact). After weaning, GFP- and FATP1-mice were fed a chow or high-fat diet. Recombinant adenovirus is a widely used delivery vector for both gene therapy and functional studies [34]. Adenovirus gene delivery, by injection into hindlimbs of immunologically naive newborn rodents, results in skeletal muscle-specific expression of the transgene [35], and persistent transgene expression [36]–[38], up to 8 months after gene delivery [37]. Here, upon completion of the study (15- to 16-week-old mice), FATP1 mRNA levels were determined in gastrocnemius muscles. No gender differences were observed in the expression of the endogenous FATP1 gene in chow-fed GFP-mice (data not shown). FATP1 mRNA content in female GFP-mice was higher (2.1-fold) in those maintained on a high-fat diet compared to those (male and female) fed chow (Figure 3A). FATP1 mRNA levels were increased in FATP1-mice compared to GFP-mice in both chow (3.8-fold) and high-fat (6.9-fold) diet subgroups. FATP1 protein content was also increased in gastrocnemius of FATP1-mice compared to GFP-mice (Figure 3B). No increased FATP1 mRNA levels were observed in soleus and tibialis anterior muscles from FATP1- compared to GFP-mice (data not shown).

It has already been shown that injection of adenovirus into hindlimbs of newborn rats results in restricted expression of the transgene in the injected muscle [35]; however, since it is known that adenoviruses have a liver tropism after entering the bloodstream [35], [39], we measured hepatic FATP1 mRNA levels in FATP1- and GFP-mice. We found that FATP1 mRNA levels were undetectable in the liver of both FATP1- and GFP-mice (females fed a chow diet). Therefore, we conclude that as expected FATP1 gene delivery was confined to hindlimb muscles and that no systemic distribution had occurred, which would have resulted in the hepatic expression of FATP1.

### Effects of FATP1 delivery to skm on blood circulating glucose and lipid metabolites and insulin in mice fed a chow or high-fat diet. Whole-body glucose and insulin tolerance

Upon completion of the study, body weight was similar in FATP1- and GFP-mice, in any of the defined gender or diet subgroups (Table 1). However, as expected, administration of the high-fat diet to females increased body weight in comparison with the chow diet, and females had a lower body weight than males on chow.

**Table 1:** Body weight, blood metabolites and insulin levels in GFP- and FATP1-mice.

	CHOW DIET				HIGH-FAT DIET			
	GFP-males	FATP1-males	GFP-females	FATP1-females	GFP- females	FATP1-females	GFP-mice	FATP1-mice
Body weight (g)	29.9±1.0	29.8±1.1	24.4±1.5^#^	24±1.1^‡^	30.4±1.4^†^	29.4±1.7^†^	-	-
Glucose (mg/dl)	120±5	133±8	111±6	115±5	141±7^*^	127.6±6	-	-
Insulin (ng/ml)	0.79±0.18	0.80±0.12	0.66±0.27	0.62±0.29	1.02±0.34	1.07±0.21	0.86±0.17	0.89±0.12
Triglycerides (mmol/l)	0.30±0.05	0.36±0.04	0.45±0.09	0.45±0.08	0.61±0.22	0.46±0.07	0.48±0.09	0.43±0.04
Fatty acids (mmol/l)	0.20±0.09	0.04±0.03	ND	ND	0.32±0.12	0.07±0.06	0.25±0.07	0.06±0.03^¥^
β-Hydroxybutyrate (mmol/l)	0.18±0.13	0.34±0.23	0.32±0.10	1.16±0.49	0.19±0.05	0.48±0.24	0.21±0.04	0.62±0.18^¥^

Body weight, blood glucose, serum insulin, triglyceride, fatty acid and β-hydroxybutyrate levels were measured in *ad libitum* fed either diet GFP- or FATP1-mice at 15- to 16-weeks of age. Data are means ± SEM of at least five samples. The significance of the Student's t test is: ^†^p<0.05 females fed a high-fat versus chow diet with the same viral treatment; ^*^p<0.01 GFP-females fed a high-fat versus chow diet; ^#^p<0.05 GFP-females versus GFP-males fed a chow diet; ^‡^p<0.005 FATP1-females versus FATP1-males fed chow; and ^¥^p<0.05 FATP1-mice versus GFP-mice irrespective of diet and gender. ND means not determined.

At this time, blood fed glucose did not differ between FATP1- and GFP-mice maintained on chow (Table 1). The high-fat diet increased glucose levels in control female mice compared to those fed chow and this effect was not observed in FATP1-mice, however no significant differences were observed between high-fat diet fed FATP1- and GFP-mice. Serum fed insulin and triglyceride levels did not differ between FATP1- and GFP-mice maintained on either chow or high-fat, there were no gender differences, nor did FATP1 exert any effect in mice grouped irrespective of diet and gender (Table 1). Moreover, the high-fat diet had no significant effect on either insulin or triglycerides, presumably due to the fact that mice were fed either diet just after weaning for a limited period of 12 to 13 weeks.

Remarkably, in the FATP1- compared to the GFP-mice grouped irrespective of diet and gender, serum fed fatty acid levels were lower, and diet had no significant effect. Since fatty acid catabolism is interrelated to ketone body metabolism, we thus assessed ketonemia, which can be determined by measuring the levels of β-hydroxybutyrate that accounts for approximately 75% of the ketone bodies in serum and during ketosis increases even more than the other two ketoacids, acetoacetate and acetone [40]. We found that β-hydroxybutyrate levels were higher in FATP1- versus GFP-mice, whereas diet and gender had no significant effect (Table 1). Changing tendencies, i.e. about 1.9- to 3.6-fold increment for β-hydroxybutyrate, which did not reach statistical significance, were observed for each of the FATP1-mice subgroups defined by gender or diet relative to mated GFP-mice.

**Figure 4:**
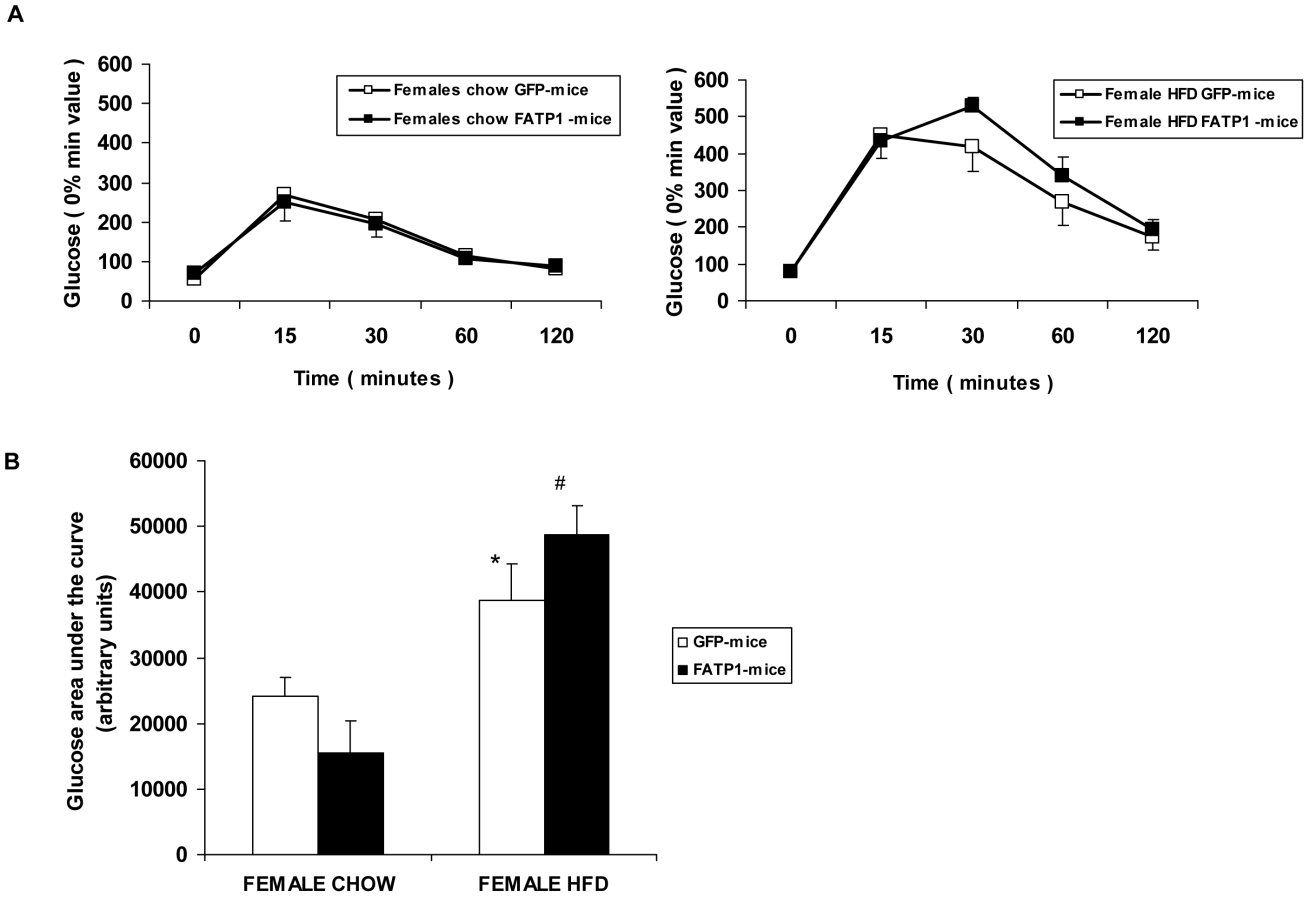
Glucose tolerance tests in GFP- and FATP1-mice. Blood glucose levels were measured before (0 min) and after glucose injection, several times up to 120 min, in GFP- or FATP1-mice fed as stated, chow or a high-fat diet (HFD). (A) Glucose data are shown and are means ±SD of five animals. (B) Glucose areas under the curve are shown and are means ± SD of five animals. The significance of the Student's t test is: *p<0.05 and ^#^p<0.001 within FATP1- or GFP-mice fed a HFD versus chow.

No differences were observed between FATP1- and GFP-mice for fasted blood glucose levels in either diet conditions (Figure 4A). Moreover, no differences in glucose tolerance were associated with FATP1 overexpression in animals fed chow or high-fat (Figure 4A), even though glucose tolerance was impaired by the high-fat diet, as shown by the elevated area under the curve for glucose within both FATP1- and GFP-mice (Figure 4B). Whole-body insulin tolerance was also unaffected by FATP1 gene delivery in either chow- or high-fat-fed females (data not shown).

### Triglyceride skm accumulation is reduced by delivery of FATP1 in mice

To test the effects of FATP1 on triglyceride tissue accumulation, triglyceride content was quantified in the gastrocnemius muscle, liver and white adipose tissue in mice fed a chow or high-fat diet (Table 2). In gastrocnemius muscle, triglyceride content was unaffected by diet and gender, and it was lower in the FATP1-mice group compared to GFP-mice group irrespective of diet and gender. No changes due to FATP1 overexpression were observed in the liver or white adipose tissue triglyceride content, in any of the studied gender or diet subgroups or irrespective of these conditions. However, as anticipated, feeding females a high-fat diet increased the triglyceride content in the liver and adipose tissue. In addition, gender differences were observed in chow-fed mice.

**Table 2:** Triglyceride levels in skm, liver and adipose tissue of GFP- and FATP1-mice.

	CHOW DIET				HIGH-FAT DIET			
TRIGLYCERIDES	GFP-males	FATP1-males	GFP-females	FATP1-females	GFP-females	FATP1-females	GFP-mice	FATP1-mice
Gastrocnemius(nmol/mg prot)	75.2±12.3	47.7±5.1	76.6±11.9	62.8±15.2	74.1±12.1	56.5±7.2	74.88±6.44	54.14±5.02^¥^
Liver(nmol/mg prot)	83.8±20.9	83.3±11.4	143±18^*^	153±22^#^	385±39^††^	391± 36^‡‡^	-	-
White adipose tissue (µmol/mg prot)	4.18±0.54	3.26±0.47	1.42±0.11^**^	1.49±0.16^#^	2.77±0.49^†^	2.58±0.35^‡^	-	-

Triglyceride levels were measured in extracts from the gastrocnemius muscle, liver and white adipose tissue of *ad libitum* fed GFP- or FATP1-mice. Data are means ± SEM of at least four samples. The significance of the Student's t test is: ^*^p<0.05 and ^**^p<0.001 female versus male GFP-mice fed chow; ^#^p<0.01 female versus male FATP1-mice fed chow; ^†^p<0.05 and ^††^p<0.001 female GFP-mice fed high-fat versus chow; ^‡^p<0.05 and ^‡‡^p<0.001 female FATP1-mice fed high-fat versus chow; and ^¥^p<0.05 FATP1- versus GFP-mice irrespective of diet and gender.

### Regulation of Hmgcs2 and *Oxct1* expression in mouse skm by FATP1 overexpression

**Figure 5:**
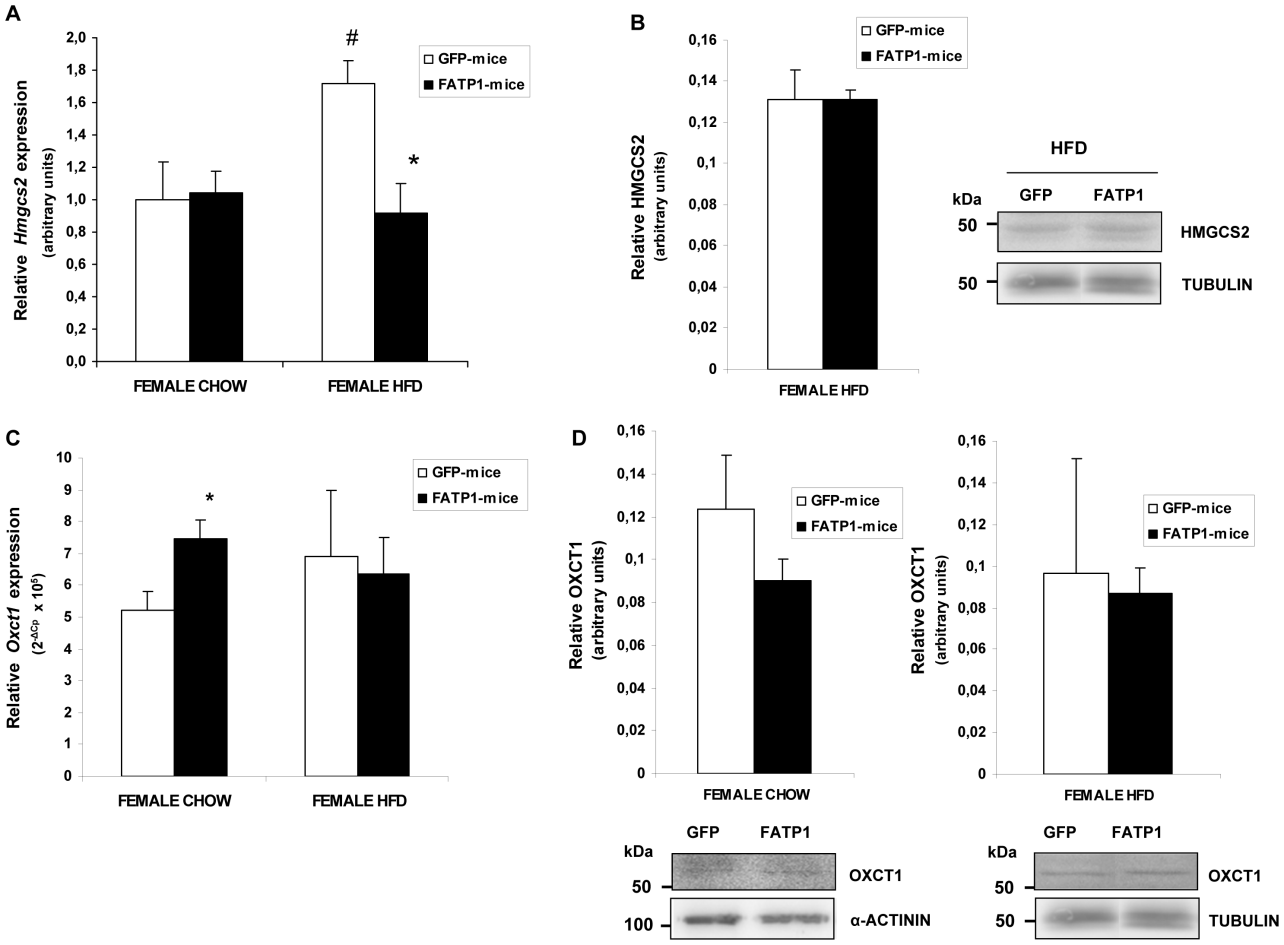
Muscle *Hmgcs2* and *Oxct1* expression in GFP- and FATP1-mice. (A) The *Hmgcs2* mRNA levels relative to *Actb* and (C) *Oxct1* mRNA levels relative to *Rn18s* were analyzed in the gastrocnemius muscles of GFP- and FATP1-female mice fed a chow or high-fat diet (HFD). (A,C) The significance of the Student's t test is: *p<0.05 FATP1- versus GFP-female mice fed on the same diet; ^#^p<0.05 control mice fed a HFD versus chow. *Hmgcs2* data are mean values of 2^−ΔCT^ ± SEM in arbitrary units from four to six samples performed in duplicate. *Oxct1* data are mean values of 2^−ΔCp^×10^5^ ± SEM from four to six samples performed in quadruplicate. (B) The HMGCS2 and (D) OXCT1 protein levels relative to tubulin or α-actinin levels were analysed. A representative image is shown and bands were quantified. Data is the ratio of intensities of bands expressed as a percentage of control and is the mean ± SEM of at least four samples performed in duplicate.

An analysis was conducted to assess whether higher levels of circulating ketone bodies in FATP1-mice were linked to the regulation of key genes of ketone body metabolism, in skm. We explored the mRNA levels of *Hmgcs2* encoding the mitochondrial protein 3-hydroxy-3-methylglutaryl-Coenzyme A synthase 2 (HMGCS2), which is the rate-limiting enzyme of ketogenesis using as primary substrates acetoacetyl-CoA and acetyl-CoA; and those of *Oxct1* encoding the mitochondrial protein succinyl-CoA:3-oxoacid-CoA transferase (SCOT/OXCT1), an enzyme that reversibly catalyzes the conversion between acetoacetate and acetoacetyl-CoA and can mediate ketone body oxidation and also ketogenesis [41]. We found that *Hmgcs2* mRNA levels (Figure 5A) were similar in the gastrocnemius muscle of chow-fed FATP1- and GFP-mice. However, *Hmgcs2* expression was enhanced by the high-fat compared to chow diet in GFP-mice, and this rise was abolished following FATP1 gene delivery. Nevertheless, no differences in HMGCS2 protein content between high-fat diet fed GFP- and FATP1-mice were observed (Figure 5B). *Oxct1* mRNA levels (Figure 5C) were slightly and significantly upregulated by FATP1 in the gastrocnemius muscle of the chow-fed female group, but not in the high fat-fed group compared to mated GFP-mice. The high-fat diet did not change *Oxct1* expression in skm, however, and as previously described [42], it reduced *Oxct1* mRNA levels (3.5-fold, p<0.05) in the heart of control mice. OXCT1 protein levels were not changed by FATP1 overexpression in either diet condition (Figure 5D).

### *Overexpression of FATP1 does not change* β-hydroxybutyrate levels in mouse skm and liver

We then determined whether overexpression of FATP1 in skm and the associated increase in circulating β-hydroxybutyrate was accompanied by altered levels of β-hydroxybutyrate in either skm or liver in mice fed a high-fat-diet. In the gastrocnemius muscle, and tibialis anterior (in which FATP1 was not overexpressed), β-hydroxybutyrate levels were not changed in FATP1- versus GFP-mice (Table 3); nor did β-hydroxybutyrate levels in the liver differ between these two mouse groups (Table 3). In addition, no differences in β-hydroxybutyrate content in the liver of chow-fed female mice were associated with FATP1 gene delivery (GFP-mice 1.08±0.10 nmol/mg wet weight and FATP1-mice 1.10±0.09 nmol/mg wet weight). Nevertheless, as expected hepatic levels of β-hydroxybutyrate were lower (p<0.01) in female mice fed chow versus high-fat irrespective of GFP or FATP1 condition, reflecting the ketogenic effect of the high-fat diet.

**Table 3:** β-hydroxybutyrate levels in skm and liver of high-fat diet fed GFP- and FATP1-mice.

β-HYDROXYBUTYRATE(nmol/mg wet weight)	GFP	FATP1
Gastrocnemius	0.80±0	1.10±0.22
Tibialis	1.32±0.04	1.37±0.05
Liver	1.31±0.05	1.54±0.12

β-hydroxybutyrate levels were measured in extracts from the gastrocnemius and tibialis anterior muscle and the liver of GFP- and FATP1-female mice fed high-fat. Data are mean values ± SEM from four to five samples.

### Regulation of active PDH levels and *Pdk4* expression in mouse skm by FATP1 overexpression

**Figure 6:**
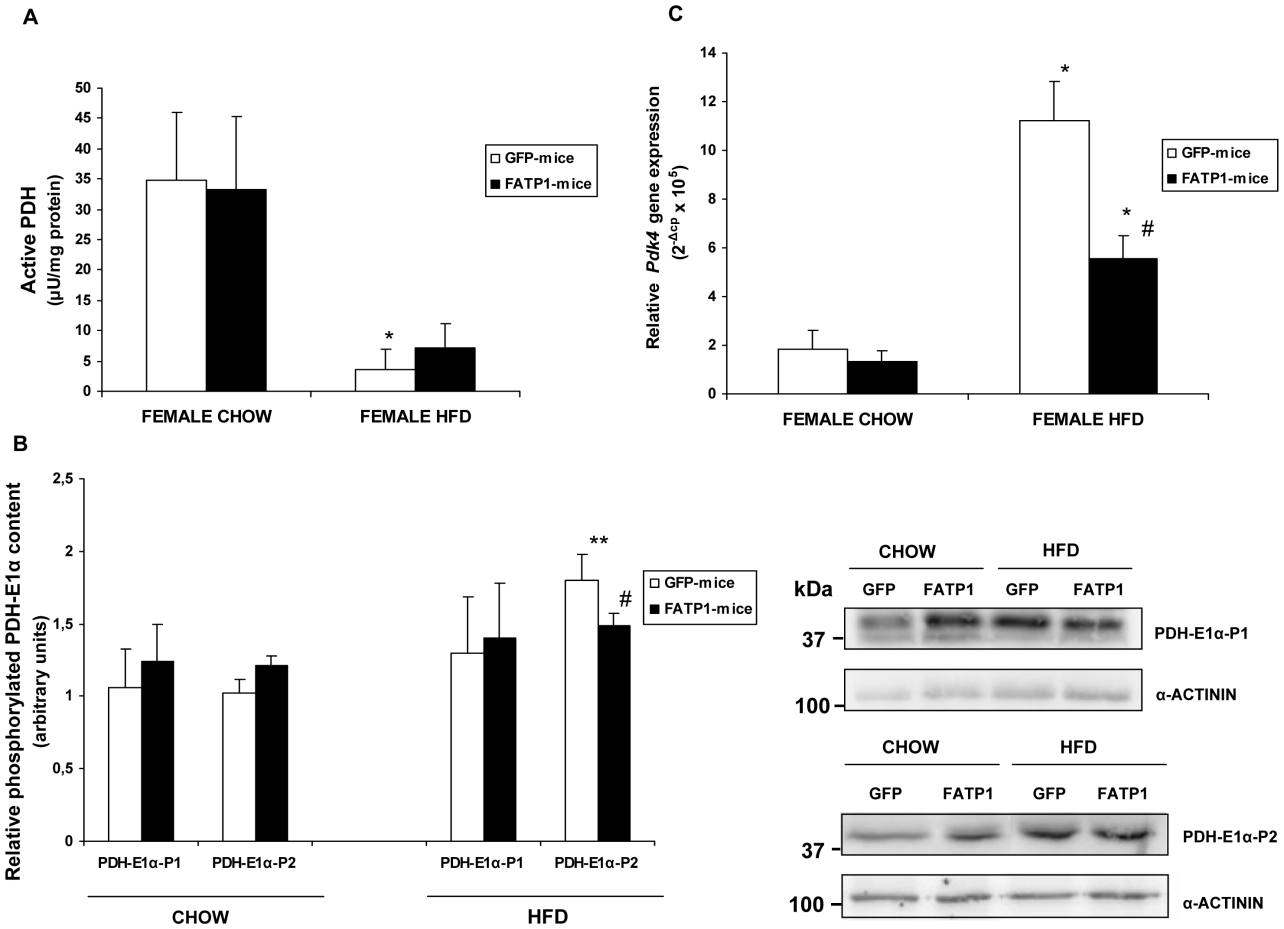
PDH levels and *Pdk4* expression in skm of GFP- and FATP1-mice. (A) Active PDH levels and (B) levels of phosphorylated PDH-E1α at sites 1 (P1) and 2 (P2) relative to α-actinin levels, were measured in extracts from the gastrocnemius muscle of GFP- and FATP1- female mice fed a chow or high-fat diet (HFD). Data are mean values ± SEM from at least four samples. The significance of the Student's t test is *p<0.05 and **p<0.01 in GFP-mice fed a high-fat versus chow diet; and ^#^p<0.05 FATP1-mice fed high-fat versus FATP1-mice fed chow (C) *Pdk4* expression was analyzed in the gastrocnemius muscle of female GFP- and FATP1-mice fed as indicated.Data are mean values of 2^−ΔCp^×10^5^ ± SEM from four to eight samples performed in duplicate. The significance of the Student's t test is: *p<0.01 in mice fed high-fat versus chow within the same GFP- or FATP1-condition; and ^#^p<0.05 FATP1-mice versus GFP-mice fed high-fat.

We have previously shown [12] that FATP1 overexpression, in cultured muscle cells, activates PDH, which is a rate-limiting step in glucose oxidation [43]. We therefore tested whether FATP1 regulated PDH activity in mouse skm (Figure 6A). We found that, in chow-fed female mice, the levels of the active form of the PDH complex were unchanged by FATP1 overexpression. Feeding with a high-fat diet reduced by 9.8-fold the activity of the active form of PDH in control mice, and FATP1 overexpression lessened this reduction although did not reach statistical significance. The PDH complex is formed by three catalytic components, namely pyruvate decarboxylase (E1, a tetramer of 2α and 2β subunits), dihydrolipoamide acetyltransferase (E2) and dihydrolipoamide dehydrogenase (E3), together with the E3-binding protein (E3BP). PDH-E1 is the rate-limiting step in the PDH complex and its activity is negatively regulated by phosphorylation of the E1α subunit at three phosphorylation sites: site 1(Ser^264^), site 2 (Ser^271^) and site 3 (Ser^203^) [43]. We analysed the levels of phosphorylated PDH-E1α at sites 1 and 2 relative to α-actinin (Figure 6B). We found that the content of phosphorylated PDH-E1α subunit at site 2 was significantly increased by the high-fat diet in control mice, whereas no significant change was observed at site 1. The diet-induced increase in the level of phosphorylated PDH-E1α at site 2, toward which pyruvate dehydrogenase kinase (PDK) 4 exhibits a much higher activity compared to PDK1, PDK2 and PDK3 [43], was correlated to the reduction in active PDH. However, to confirm this regulation the ratio of phosphorylated versus total PDH-E1α should be determined. On the other hand, changes in the phosphorylation of PDH-E1α sites 1 and 2 are not consistently reflected in active PDH activity changes in muscle and the participation of other covalent undefined modifications has been suggested [28]. No significant effect of FATP1 was observed in the phosphorylation of PDH-E1α at sites 1 and 2 in chow-fed mice, even though the high-fat diet-induced increase in site 2 phosphorylation tended to be attenuated in FATP1-mice, in association with a less pronounced fall in PDH activity.

We also analyzed whether pyruvate dehydrogenase kinase 4 (PDK4) mRNA levels in mouse skm were altered by FATP1. There was no change in *Pdk4* expression due to FATP1 gene delivery in mice fed a chow diet, both when separated by gender (Figure 6C) or grouped together (data not shown). However, the high-fat diet upregulated *Pdk4* mRNA levels and FATP1 overexpression partially counteracted this increase (Figure 6C).

### Oxidative disposal of palmitate and β-hydroxybutyrate is altered by FATP1 overexpression in mouse skm

**Figure 7:**
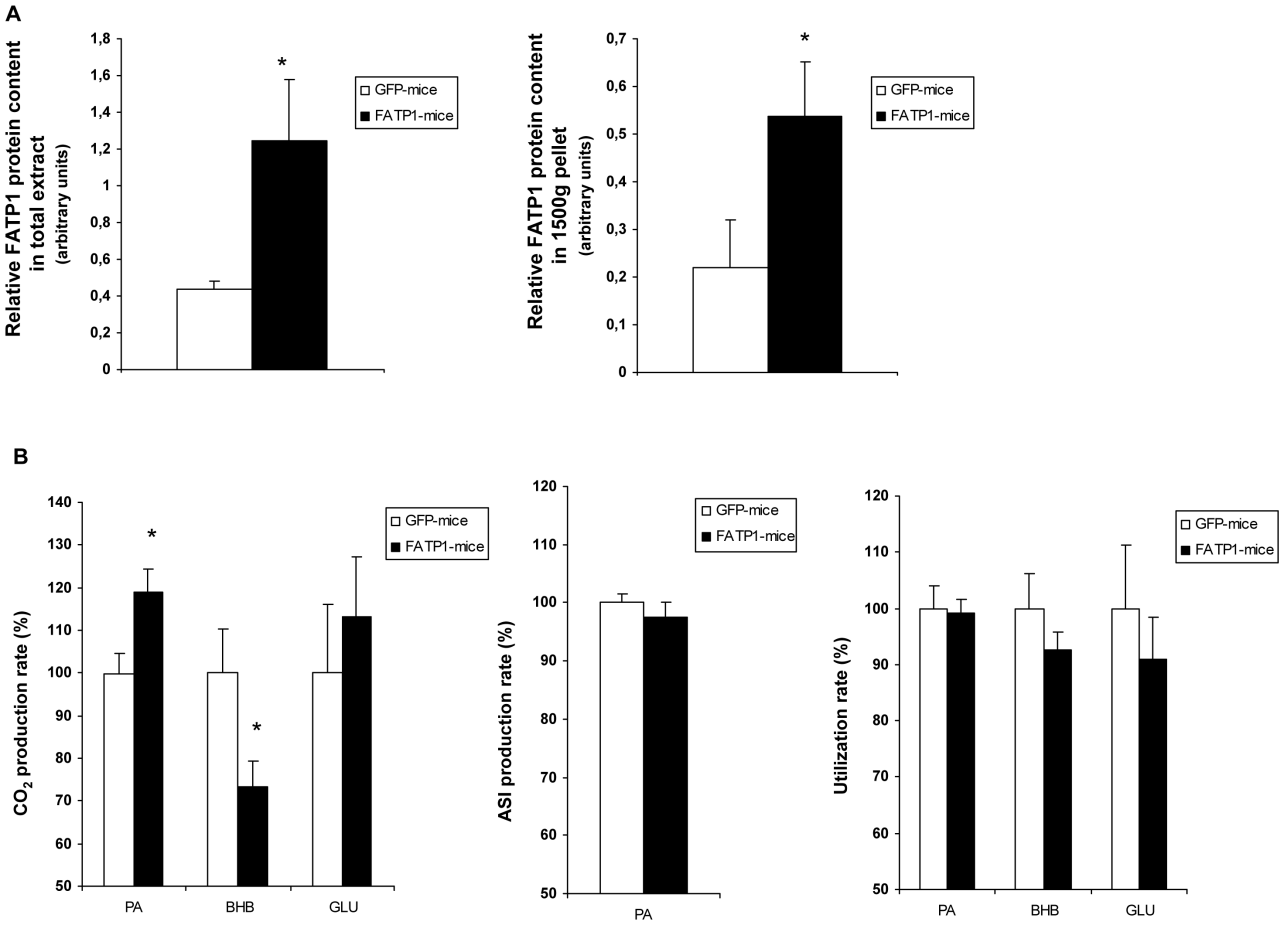
Effects of FATP1 overexpression on gastrocnemius muscle palmitate, β-hydroxybutyrate and glucose oxidation. Gastrocnemius muscle strips were prepared from pGFP- or pFATP1-electropored mice. (A) The FATP1 protein levels relative to α-tubulin levels were determined in total extracts and the 1500 *g* pellet fraction (20 µg of protein) by immunoblotting. Bands were quantified with a LAS-3000 (FujiFilm). Data is the ratio of intensities of bands in arbitrary units and is the mean ± SEM of at least five samples. (B) Muscle strips were incubated for 4 h with radioactively labelled palmitate (PA), β-hydroxybutyrate (BHB) or glucose (GLU). At the end of this period, substrate utilization rate, ^14^CO_2_ production rate (and [^14^C]acid-soluble intermediate metabolites (ASI) production rate in palmitate-incubated strips) were determined. Data are expressed as a percentage of control and are the means ± SEM of seven muscle samples. (A,B) The significance of the Student's t test versus controls is *p<0.05.

Our data suggested that FATP1 overexpression in gastrocnemius muscle could alter the oxidative disposal of fatty acids and ketone bodies. To test this, we electropored pFATP1 or pGFP plasmids into the gastrocnemius muscle of mice. Ten days after transfection, gastrocnemius muscle strips were obtained. Increased FATP1 protein content was shown in muscle strips from mice electropored with pFATP1 in comparison to pGFP-electropored mice, in both total extracts (2.8-fold) and the 1500 *g* pellet fraction enriched in mitochondria (2.4-fold), suggesting that the transfected protein mimics the endogenous protein localization (Figure 7A). In isolated muscle strips, the rate of oxidation and utilization of radioactively labelled 0.5 mM palmitate, 2 mM β-hydroxybutyrate or 10 mM glucose was determined (Figure 7B). FATP1 overexpression increased by 19% [1-^14^C]palmitate oxidation to ^14^CO_2_ (rate in controls 0.88±0.04 pmol/mg tissue x h). The production of ^14^CO_2_ from [3-^14^C]β-hydroxybutyrate was, in contrast, reduced by 27% (rate in controls 5.43±0.60 pmol/mg tissue x h) and that from [U-^14^C]glucose unchanged (rate in controls 9.64±1.55 pmol/mg tissue x h). The production of [^14^C]acid-soluble intermediate metabolites due to incomplete [1-^14^C]palmitate oxidation (including acetyl-CoA, acetyl-carnitine, intermediates of Krebs cycle and ketone bodies) was unchanged by FATP1 (rate in controls 89.2±1.4 pmol/mg tissue x h). Finally, no significant changes were observed in the consumption of either substrate [1-^14^C]-palmitate (5.52±0.21 nmol/mg tissue x h in controls), p-[3-^14^C]hydroxybutyrate (10.9±0.7 nmol/mg tissue x h in controls) or [U-^14^C]glucose (62.6±7.1 nmol/mg tissue x h in controls), even though a reduction tendency was observed for the ketone body and glucose. In control gastrocnemius (pGFP-electropored), the ratio of ^14^CO_2_ production to labelled substrate consumption was about 3-fold greater for the ketone body than for palmitate and glucose, in accordance to the more diversified metabolism of the latter two substrates.

## Discussion

FATP1 is considered a mediator of fatty acid import in skm, but its prevailing intracellular localization remains under debate. Our results strengthen the evidence for its mitochondrial localization, in both skm tissue and cultured cells. We show that, in mouse skm, the endogenous FATP1 protein was mostly present in mitochondrial tissue fractions and in purified mitochondria; and that transfected FATP1 increased FATP1 protein content in mitochondrial fractions. A previous study, in isolated mouse soleus muscle, revealed a marked intracellular localization of the endogenous FATP1 protein in a vesicle population that was not defined [14]. However, no support for the mitochondrial localization was obtained in FATP1-transfected rat muscle, in which there was no increase in FATP1 protein on mitochondria [16]. Moreover, by applying digitonin followed by differential centrifugation to purified mitochondria, we provide insight into FATP1 submitochondrial localization. FATP1 was detected in the outer membrane and intermembrane fractions rather than in the inner membrane plus matrix fraction. Considering that FATP1 is an integral membrane protein with one transmembrane domain [4], we suggest that it may be an integral outer membrane protein. Finally, using immunocytochemical localization via immunogold electron microscopy, we provide herein evidence for the mitochondrial localization of the FATP1-GFP protein in C2C12 muscle cells. Consistent with our results here, the endogenous FATP1 protein in murine and human cultured skm cells is present in mitochondria-enriched fractions [12], and the fusion protein FATP1-GFP colocalizes with mitochondrial markers in C2C12 [12] and L6E9 [13] muscle cells. Indeed, a bioinformatic analysis predicts FATP1 import into mitochondria, as well as that of two other members of the FATP family, FATP2 and FATP4; and using protein cross-linking, a number of mitochondrial and mitochondrially associated proteins that bind FATP1 in 3T3-L1 adipocytes have been identified [9]. It should be noted however, that the mitochondrial localization of FATP1 in adipocytes remains controversial, since no significant overlap of FATP1 with mitochondria was observed in another study [10]. On the other hand, we show that FATP1 was also present in an enriched plasma membrane fraction from mouse gastrocnemius muscle, consistent with the description of its localization in sarcolemma of lower hindlimb rat muscles [14], [15] and plasma membrane of 3T3-L1 adipocytes [3], [6], [7]. This type of dual distribution has been observed for VDAC, which is an abundant protein in the outer mitochondrial membrane, but is also present in the plasma membrane [33].

The differential effect of FATP1 overexpression on fatty acid metabolism has been reported in cultured skm cells, in which it consistently promotes fatty acid diversion towards triglyceride storage [11], [13], and in mouse skm tissue, in which FATP1 targets fatty acids to oxidation but not triglyceride accumulation [16]. To gain further insight into skm FATP1 biochemical role *in vivo*, we used adenovirus to deliver the FATP1 gene into hindlimb muscles of mice that were then fed a chow or a high-fat diet after weaning. Mouse body weight was not altered by FATP1 overexpression in either feeding condition; consistently with the lack of effect on body weight that is observed in muscle-specific FATP1-overexpressing transgenic mice compared to wild-type mice, fed either a chow or a high-fat diet [16]. Serum fed triglycerides levels were unchanged in FATP1- compared to GFP-mice regardless of diet; however, fatty acid levels were reduced and β-hydroxybutyrate levels were increased. The enhanced systemic fatty acid disposal in FATP1-mice is concordant with the proven capacity of overexpressed FATP1 to mediate fatty acid uptake, as shown in cultured skm cells [11] and tissue [16]. Here, intramuscular triglyceride levels were reduced by FATP1 overexpression irrespective of diet, and there were no diet-associated changes, as already observed in rat muscle [44]. Our data diverge from that in muscle-specific FATP1-overexpressing transgenic mice [16], which show unaltered non-fasting serum triglyceride and NEFA levels compared to wild-type mice, before or after a high-fat diet, and unaltered intramuscular triglyceride content on a high-fat diet. We suggest that this differential lipid metabolic pattern may be due to distinct metabolic adaptations to muscle-directed transgenic versus skm-directed adenoviral postnatal gene transfer of FATP1. On the other hand, in our FATP1-mice, triglyceride content in both the liver and white adipose tissue was unchanged compared to GFP-mice, indicating no redistribution of tissue triglyceride stores. These data contrast with that reported for the abrogation of FATP1 in mice, which causes a redistribution of lipids from adipose and muscle tissue to the liver, in both high- and low-fat-fed animals [14], a discrepancy that may be explained by the key role of FATP1 expressed in adipose tissue in whole body lipid metabolism. In summary, here we show that *in vivo* FATP1 reduced both serum fatty acids and intramuscular triglyceride content, suggesting it enhanced fatty acid oxidative disposal, as shown by others in skeletal [16] and cardiac [45] muscle. In contrast, this effect is limited [13] or not observed [11] in cultured myotubes, where FATP1 directs imported fatty acids mainly towards storage in triglycerides.

A striking finding of this work is the increment in serum β-hydroxybutyrate levels associated with muscle FATP1 overexpression. The skm, as well as cardiac muscle, has a high capacity to metabolize ketone bodies and it is widely accepted that the majority of used ketone bodies originate from the liver and are extracted from the circulation [41]. However, ketogenesis, which occurs mostly from β-oxidation-derived acetyl-CoA [41], has been demonstrated in isolated mouse skm mitochondria, i.e. production of β-hydroxybutyrate from palmitate [46]. The key ketogenic gene encoding mitochondrial HMGCS2 is expressed at low levels in human skm at the transcript [47] and protein [48] level. However, the enzymes involved in ketone body oxidation, OXCT1 and mitochondrial thiolase, catalyze reversible reactions and, in consequence, tissues that oxidize ketone bodies have the potential to synthesize them [41]. In this regard, evidence has been obtained for the production of ketone bodies from palmitate in perfused rat heart, in a process mediated by the reversal of OXCT1 reaction [49]. Here, we found upregulation of *Hmgcs2* by the high-fat diet in skm, in agreement with the observation that *Hmgcs2* is upregulated by fatty acids in HeLa cells [50] and L6 myotubes [51]. FATP1 overexpression did not alter *Hmgcs2* mRNA levels in muscle from chow-fed mice, but prevented the rise caused by the high-fat diet, although no changes were observed at the HMGCS2 protein level. This suggested that increased ketonemia in FATP1-mice is not due to upregulation of the ketogenic gene *Hmgcs2* in skm. We thus reasoned that it could be due to regulation of OXCT1, a required enzyme for ketone body oxidation [52], which can be involved in ketogenesis [49]. We found that skm *Oxct1* expression was enhanced by FATP1 in mice fed chow, but unchanged in those fed a high-fat diet, while OXCT1 protein levels were unchanged in both diet conditions. Furthermore, we observed no repression of *Oxct1* by the high-fat diet in skm, in contrast to the repressing effect detected in mouse heart, as previously described [42]. We thus conclude that the diet-independent effect of FATP1 on ketone body disposal is not linked to *Oxct1* regulation in skm. Furthermore, we found no substantial intramuscular accumulation of β-hydroxybutyrate, associated to hyperketonemia, in high-fat-fed FATP1-mice compared to controls. Consequently hyperketonemia in FATP1-mice could be secondary to the FATP1-mediated enhanced oxidation of fatty acids in skm. The latter could impair the extraction and/or use of circulating ketone bodies, due to their competition for oxidation, i.e. inhibition of acetoacetate oxidation by fatty acids as shown in rat kidney cortex [53]; or augment ketogenesis, as a result of the high rate of fatty acid oxidation. We show that indeed FATP1 overexpression enhanced palmitate oxidation to CO_2_, but not to acid-soluble intermediate metabolites (which include ketone bodies), while it inhibited CO_2_ production from β-hydroxybutyrate, in isolated mouse gastrocnemius strips. In addition, in isolated mitochondria from FATP1-overexpressing L6E9 myotubes, palmitoyl-CoA oxidation to CO_2_ but not to acid-soluble metabolites is enhanced [13]. Therefore, we conclude that the ketone body sparing effect of skm FATP1 is due to inhibition of ketone body oxidation rather than to ketogenesis.

Circulating fed glucose and insulin levels were unchanged in FATP1- compared to GFP-mice fed either diet; and the same occurred with whole body glucose and insulin tolerance. Likewise, muscle-specific FATP1-overexpressing transgenic mice [16] have unaltered blood non-fasting glucose and insulin levels, before and after a high-fat diet, and unchanged glucose and insulin tolerance on a high-fat diet. All these data support that FATP1 overexpression in muscle does not predispose to high-fat diet-induced insulin resistance. In apparent contradiction, FATP1 gene ablation in mice prevents impaired insulin sensing in high-fat-fed animals [14], [18]. Presumably, this discrepancy between muscle-restricted and genetic manipulation of endogenous FATP1 gene expression is due to a different contribution of adipose tissue FATP1 versus muscle FATP1 to whole body metabolism.

In cultured myotubes, FATP1 strongly stimulates glucose use and complete oxidation of glucose and lactate to CO_2_, and activates PDH [12]. In this study, FATP1 had no effect on the levels of the active form of the PDH complex in skm of mice fed chow, nor did it enhance CO_2_ production from glucose in isolated gastrocnemius strips. Thus, we conclude that FATP1 in skm does not exert an stimulatory effect on glucose use or PDH activity, in contrast to *in vitro* observations [12]. However, FATP1 lessened the strong reduction in PDH activation caused by the high-fat diet, a diet effect previously shown in rats [54]. Furthermore, FATP1 tended to attenuate the high-fat diet-induced increase in PDH-E1α site 2 phosphorylation, which is known to negatively regulate the rate-limiting PDH-E1 [43]. PDH is phosphorylated and inactivated by PDKs. The PDK4 gene is highly expressed in skm [55], is upregulated by high-fat diets [54], [56], [57] and has been proposed to underlie PDH inactivation in response to high-fat diets [54]. We found that FATP1 had no effect on skm *Pdk4* mRNA levels, in mice fed chow, in keeping with the lack of effect shown in cultured myotubes [12]; but it did significantly counteract to some degree *Pdk4* upregulation by the high-fat diet. Hence, FATP1 curbs high-fat diet induction of two genes regulated through a peroxisome proliferator activated receptor (PPAR)-alpha mechanism, namely *Hmgcs2* [50] and *Pdk4* [55]; whereas the mRNA level of *Oxct1*, which is not clearly regulated by PPAR-alpha [42], is differently affected by FATP1. It is possible that by stimulating fatty acid consumption FATP1 alters the PPAR-alpha-mediated pathway. In connection with this, in liver, 1-palmitoyl-2-oleoyl-sn-glycerol-3-phosphocholine (which is generated by the novo lipid biosynthesis) has been identified as an endogenous ligand of PPAR-alpha [58].

In summary, our data provide further evidence for the localization of FATP1 in mitochondria in both skm tissue and cultured cells, supporting the notion that FATP1 can exert an indirect effect on fatty acid use, likely based on its metabolic trapping activity, which could occur in different intracellular compartments including mitochondria. Moreover, our findings indicate that, in skm tissue, FATP1 enhances the use of fatty acids, which may be extracted from the circulation or mobilized from intramuscular triglyceride stores, and promotes fatty acid oxidation; effects that are known to be protective for insulin sensitivity in skeletal muscle [59], but pathogenic in cardiac muscle [45]. Concomitantly, FATP1 attenuates the effects of the high-fat diet on the skm mRNA levels of the PPAR-alpha target genes *Hmgcs2* and *Pdk4*. Thus, suggesting that skm FATP1 protects against lipotoxicity and in consequence diet-induced metabolic dysregulation. However, FATP1 increases irrespective of diet circulating ketone bodies, an effect that is unrelated to the regulation of expression of the crucial ketone metabolic genes *Hmgcs2* and *Oxct1* in skm, and likely due to the sparing of ketone body oxidation secondary to the enhanced fatty acid oxidation.
